# Supplementary material for: Tumor-Specific miRNA Signatures in Combination with CA19-9 for Liquid Biopsy-Based Detection of PDAC
Source: Int J Mol Sci. 2021 Dec 19;22(24):13621. doi: 10.3390/ijms222413621 (PMC8703833; doi:10.3390/ijms222413621)
Supplement: Supplementary file 1 [file ijms-22-13621-s001.zip › ijms-1488825-supplementary.pdf]

International Journal of
Molecular Sciences

MDPI

Supplementary information

Tumor-specific miRNA signatures in combination with CA19-9 for liquid biopsy-based detection of PDAC

Min Woo Kim [1,‡], Hani Koh [2,‡], Jee Ye Kim [1], Suji Lee [1], Hyojung Lee [1], Young Kim [1], Ho Kyoung Hwang [2,*], and Seung Il Kim [1,*]

[1] Division of Breast Surgery, Department of Surgery, Yonsei University College of Medicine, Korea
[2] Division of Hepatobiliary and Pancreatic Surgery, Department of Surgery, Yonsei University College of Medicine, Korea
‡ These authors contributed equally to this work.
* Correspondence: Seung Il Kim, SKIM@yuhs.ac; Ho Kyoung Hwang, DRHHK@yuhs.ac

Abstract: Pancreatic ductal adenocarcinoma (PDAC) is considered one of the most aggressive malignancies and has high mortality and poor survival rates. Therefore, there is an urgent need to discover non-invasive biomarkers for early detection before PDAC reaches the incurable stage. We hypothesized that liquid biopsy of PDAC-derived extracellular vesicles (PDEs) containing abundant microRNAs (miRNAs) could be used for early diagnosis of PDAC because they can be selectively enriched and because they are biologically stable. We isolated PDEs by immuno-capture using magnetic beads, and we identified 13 miRNA candidates in 20 pancreatic cancer patients and 20 normal controls. We found that the expression of five miRNAs, including miR-10b, miR-16, miR-155, miR-429, and miR-1290, was markedly higher in PDEs. Furthermore, the miRNA signatures along with serum carbohydrate antigen 19-9 (CA19-9) were optimized by logistic regression, and the miRNA signature and CA19-9 combination markers (CMs) were effective at differentiating PDAC patients from normal controls. As a result, the CMs represented a high sensitivity (AUC, 0.964; sensitivity, 100%; specificity, 80%) and a high specificity (AUC, 0.962; sensitivity, 85.71%; specificity, 100%). These findings suggest that five miRNAs expressed in PDEs and CA19-9 are valuable biomarkers for screening and diagnosis of pancreatic cancer by liquid biopsy.

Keywords: pancreatic cancer; diagnosis; liquid biopsy; extracellular vesicles; microRNA; CA19-9

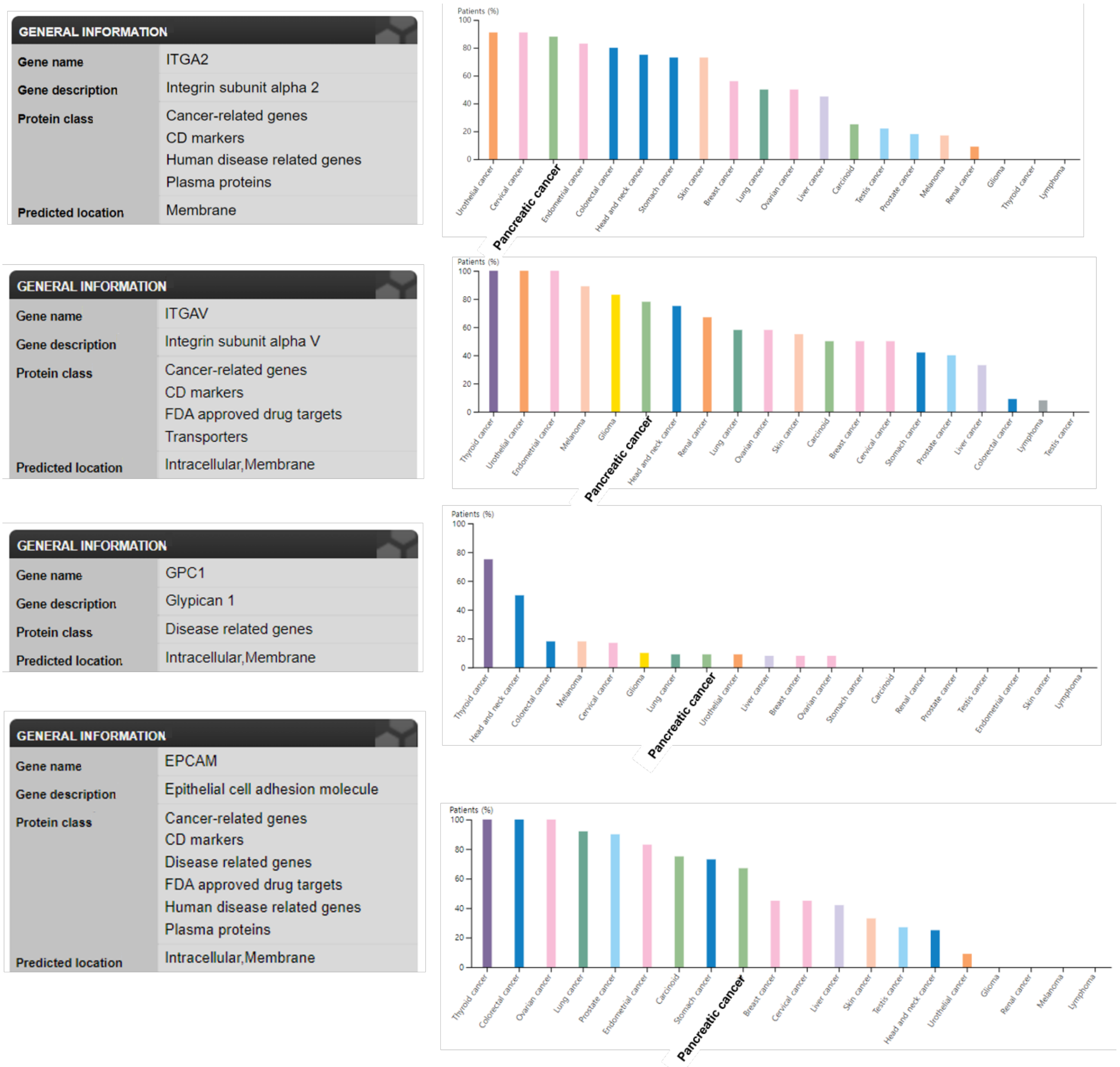

Figure S1. Candidates of pancreatic cancer-associated surface markers. For each cancer, color-coded bars indicate the percentage of patients (maximum 12 patients). (A) ITGA2 protein expression in cancer tissues. 7 of 8 patients show high/medium expression. (B) ITGAV protein expression in cancer tissues. 7 of 9 patients show high/medium expression. (C) GPC1 protein expression in cancer tissues. 1 of 11 patients show high/medium expression and 2 of 11 patients show medium/low expression. (D) EpCAM protein expression in cancer tissues. 8 of 12 patients show high/medium expression.

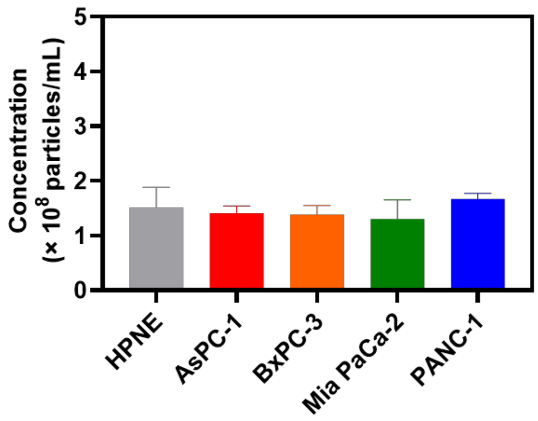

Figure S2. Measurement of EV amounts by NTA. The bar graph represents a typical analysi s of average EV concentration with standard deviation from five independent experiments.

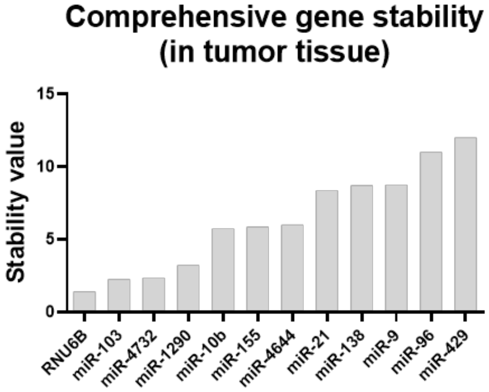

Method	1	2	3	~	10	11	12
Recommended comprehensive ranking	**RNU6B**	miR-103	miR-4732	~	miR-9	miR-96	miR-429

Figure S3. Comprehensive expression stability of 12 candidate miRNAs in pancreatic tumor tissues according to their stability value calculated by RefFinder. The top-ranking reference gene is shown in bold.

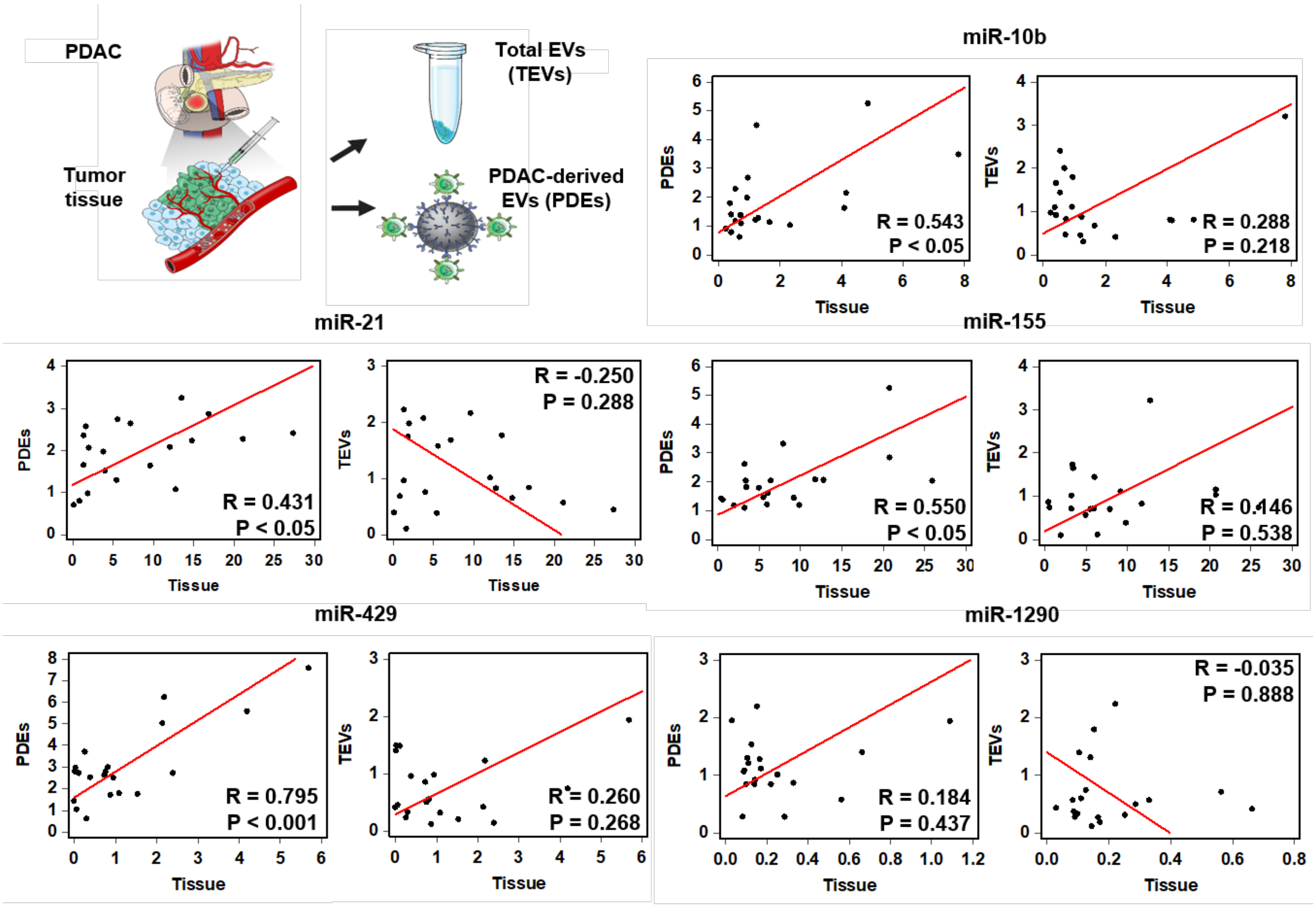

Figure S4. Correlation analyses between fold change of miRNA in tumor tissue and those in EVs. (A) Spearman's correlation coefficient analysis of between miRNAs in PDEs (y-axis in the left panel), TEVs (y-axis in the right panel), and tumor tissues (x-axis in both panels). PDEs were isolated by immunocapture using magnetic microbeads functionalized with pancreatic cancer-specific antibodies (ITGA2, ITGAV, and GPC1) and TEVs were isolated by precipitation using a total exosome isolation kit following protocols explained in the material and method section.

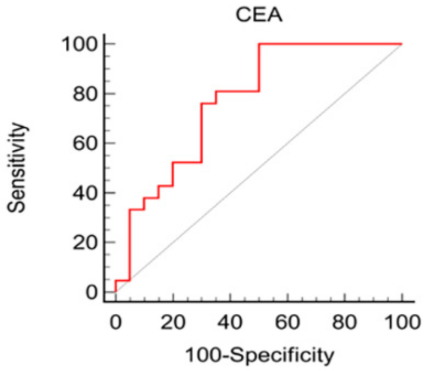

CEA

Sample size	41
Positive group [a]	21 (51.22%)
Negative group [b]	20 (48.78%)

Area under the ROC curve (AUC)

Area under the ROC curve (AUC)	0.771
Standard Error [a]	0.0758
95% Confidence interval [b]	0.614 to 0.888
z statistic	3.583
Significance level P (Area=0.5)	0.0003

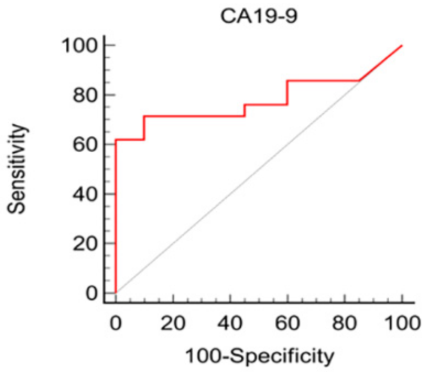

CA19-9

Sample size	41
Positive group [a]	21 (51.22%)
Negative group [b]	20 (48.78%)

Area under the ROC curve (AUC)

Area under the ROC curve (AUC)	0.78
Standard Error [a]	0.08
95% Confidence interval [b]	0.623 to 0.894
z statistic	3.497
Significance level P (Area=0.5)	0.0005

Figure S5. Receiver operating characteristic (ROC) curve analysis of conventional biomarkers CEA and CA19-9 in PDAC patients and normal controls.

Table S1. Fold changes in miRNA expression in 20 PDAC patients and 20 CL controls

	CL vs. PDAC			
**microRNAs**	**Mean FC**	**SD**	**P value**	**AUC**
**miR-21**	**1.84**	**0.81**	**< 0.001**	**0.771**
**miR-155**	**1.94**	**0.97**	**0.001**	**0.843**
**miR-429**	**3.02**	**1.75**	**< 0.001**	**0.867**
miR-96	2.13	3.59	0.465	0.507
miR-9	1.45	0.81	0.141	0.593
miR-138	1.16	0.64	0.862	0.502
miR-103	1.02	0.49	0.976	0.505
**miR-1290**	**3.61**	**4.88**	**0.029**	**0.786**
miR-4732	1.40	0.79	0.697	0.598
miR-484	1.21	0.46	0.320	0.574
**miR-10b**	**1.83**	**1.24**	**0.016**	**0.693**
miR-4644	1.05	1.41	0.317	0.631
miR-3976	N/A	N/A	N/A	N/A

International Journal of
Molecular Sciences

MDPI

Table S2. Logistic regression to evaluate the performance of miRNA multi-panels

	Combination			Logistic regression equation	Sensitivity	Specificity	AUC	SE	95% CI
1	miR_21	miR_155		-5.515+1.702*LN(miR_21)+2.368*LN(miR_155)	80.95	80	0.871	0.0577	0.730 to 0.955
2	miR_21	miR_429		-4.405+1.968*LN(miR_21)+0.959*LN(miR_429)	76.19	90	0.867	0.0604	0.724 to 0.952
3	miR_21	miR_1290		-5.983+2.742*LN(miR_21)+1.596*LN(miR_1290)	76.19	90	0.898	0.0485	0.763 to 0.970
4	miR_21	miR_10b		-5.332+2.372*LN(miR_21)+1.682*LN(miR_10b)	80.95	85	0.862	0.0633	0.718 to 0.949
5	miR_155	miR_429		-5.104+2.650*LN(miR_155)+0.758*LN(miR_429)	76.19	85	0.869	0.0549	0.727 to 0.954
6	miR_155	miR_1290		-7.28043+3.56894*LN(miR_155)+1.48034*LN(miR_1290)	76.19	85	0.91	0.0466	0.778 to 0.976
7	miR_155	miR_10b		-9.25778+4.37639*LN(miR_155)+2.40311*LN(miR_10b)	85.71	90	0.912	0.0459	0.781 to 0.978
8	miR_429	miR_1290		-4.871+1.263*LN(miR_429)+1.581*LN(miR_1290)	76.19	90	0.931	0.0389	0.806 to 0.986
9	miR_429	miR_10b		-4.846+1.422*LN(miR_429)+1.596*LN(miR_10b)	80.95	85	0.907	0.0492	0.775 to 0.975
10	miR_1290	miR_10b		-3.45636+1.23743*LN(miR_1290)+1.14198*LN(miR_10b)	66.67	90	0.81	0.0718	0.657 to 0.915
11	miR_21	miR_155	miR_429	-6.128+1.573*LN(miR_21)+1.949*LN(miR_155)+0.723*LN(miR_429)	76.19	90	0.895	0.0517	0.759 to 0.969
12	miR_21	miR_155	miR_10b	-11.53079+2.02261*LN(miR_21)+3.77918*LN(miR_155)+2.80438*LN(miR_10b)	85.71	90	0.94	0.0393	0.819 to 0.990
13	miR_21	miR_155	miR_1290	-10.98129+2.82250*LN(miR_21)+3.13933*LN(miR_155)+1.97436*LN(miR_1290)	90.48	90	0.95	0.0346	0.833 to 0.994
14	miR_21	miR_429	miR_1290	-8.523+2.840*LN(miR_21)+1.171*LN(miR_429)+1.729*LN(miR_1290)	85.71	90	0.943	0.0352	0.823 to 0.991
15	miR_21	miR_429	miR_10b	-6.69937+1.83552*LN(miR_21)+1.05953*LN(miR_429)+1.70841*LN(miR_10b)	80.95	85	0.921	0.0407	0.794 to 0.982
16	miR_21	miR_1290	miR_10b	-7.08553+2.64320*LN(miR_21)+1.52779*LN(miR_1290)+1.04969*LN(miR_10b)	85.71	90	0.91	0.0476	0.778 to 0.976
17	miR_155	miR_429	miR_1290	-8.961+3.297*LN(miR_155)+0.953*LN(miR_429)+1.713*LN(miR_1290)	76.19	90	0.943	0.0342	0.823 to 0.991

18	miR_155	miR_429	miR_10b			-10.90306+4.06792*LN(miR_155)+0.90379*LN(miR_429)+2.53101*LN(miR_10b)	90.48	85	0.936	0.0405	0.813 to 0.988
19	miR_155	miR_1290	miR_10b			-10.53068+4.16220*LN(miR_155)+1.26091*LN(miR_1290)+2.10808*LN(miR_10b)	80.95	90	0.955	0.0297	0.840 to 0.995
20	miR_429	miR_1290	miR_10b			-6.34184+1.35766*LN(miR_429)+1.34857*LN(miR_1290)+1.19637*LN(miR_10b)	85.71	90	0.938	0.0374	0.816 to 0.989
21	miR_21	miR_155	miR_429	miR_1290		-13.210+3.008*LN(miR_21)+2.891*LN(miR_155)+1.075*LN(miR_429)+2.234*LN(miR_1290)	90.48	90	0.962	0.0289	0.850 to 0.997
22	miR_21	miR_155	miR_429	miR_10b		-12.50475+1.63821*LN(miR_21)+3.55128*LN(miR_155)+0.77352*LN(miR_429)+2.90782*LN(miR_10b)	90.48	85	0.948	0.0363	0.830 to 0.993
23	miR_21	miR_155	miR_1290	miR_10b		-14.16669+2.68100*LN(miR_21)+3.77656*LN(miR_155)+1.89451*LN(miR_1290)+2.05676*LN(miR_10b)	90.48	90	0.964	0.027	0.854 to 0.998
24	miR_21	miR_429	miR_1290	miR_10b		-9.76996+2.71412*LN(miR_21)+1.19428*LN(miR_429)+1.70343*LN(miR_1290)+1.05849*LN(miR_10b)	85.71	90	0.95	0.0308	0.833 to 0.994
25	miR_155	miR_429	miR_1290	miR_10b		-11.38398+3.63583*LN(miR_155)+0.87567*LN(miR_429)+1.36606*LN(miR_1290)+1.86917*LN(miR_10b)	85.71	85	0.96	0.0264	0.847 to 0.996
26	miR_21	miR_155	miR_429	miR_1290	miR_10b	-16.53978+2.97144*LN(miR_21)+3.38367*LN(miR_155)+1.06105*LN(miR_429)+2.25739*LN(miR_1290)+2.01565*LN(miR_10b)	90.48	90	0.974	0.0202	0.869 to 0.999

International Journal of
Molecular Sciences

Table S3. Antibodies used in this research

Target	Vendor	Catalog No.	Host species
Integrin a2 (Biotinylated)	R&D Systems	BAM1233	Mouse
Integrin av (Biotinylated)	STEMCELL	60043BT	Mouse
Glypican 1 (Biotinylated)	R&D Systems	BAF4519	Goat
EpCAM (Biotinylated)	Abcam	ab79079	Mouse
CD63 (PE-Cy™7)	BD Biosciences	561982	Mouse

International Journal of
Molecular Sciences

MDPI

Table S4. Clinical information for PDAC patients and CL controls

PDAC	Age	Gender	Diagnosis	T	N	M	AJCC Stage	CA19-9	CEA
C1	54	M	PDAC	T1c	N0	M0	IA	47.3	3.27
C2	73	M	PDAC	T1a	N0	M0	IA	29.7	3.58
C3	64	M	PDAC	T1c	N0	M0	IA	38.5	7.09
C4	35	M	PDAC	T1c	N1	M0	IIB	389	2.33
C5	67	M	PDAC	T1c	N1	M0	IIB	199	6.07
C6	53	F	PDAC	T2	N1	M0	IIB	8.3	1.58
C7	60	M	PDAC	T3	N2	M0	III	297	3.42
C8	75	M	PDAC	T1c	N0	M0	IA	<2.0	4.62
C9	72	M	PDAC	T2	N0	M0	IB	91.6	4.82
C10	56	M	PDAC	T2	N0	M0	IB	92.2	2.62
C11	59	F	PDAC	T1c	N1	M0	IIB	16.1	1.62
C12	59	F	PDAC	T1c	N1	M0	IIB	<2.0	3.12
C13	62	M	PDAC	T2	N2	M0	III	761	1.62
C14	48	F	PDAC	T2	N1	M0	IIB	176	11.1
C15	52	M	PDAC	T1b	N1	M0	IIB	18.7	2.71
C16	76	M	PDAC	T1c	N0	M0	IA	6.1	2.63
C17	52	M	PDAC	T1c	N0	M0	IA	29.9	2.01
C18	78	M	PDAC: No residual carcinoma	T1a	N0	M0	IA	124	6.73
C19	75	F	PDAC	T1c	N1	M0	IIB	187	4.91
C20	57	M	PDAC	T1c	N0	M0	IA	<2.0	1.42

CL	Age	Gender	Diagnosis	T	N	M	AJCC Stage	CA19-9	CEA
N1	41	F	Chronic cholecystitis	N/A				3.9	0.81
N2	67	F	Chronic cholecystitis					8.4	0.84
N3	51	F	Chronic cholecystitis					3.7	0.87
N4	51	F	Chronic cholecystitis					<2.0	7.78
N5	66	M	Chronic cholecystitis					10.7	1.94
N6	44	M	Chronic cholecystitis					13.5	3.45
N7	64	M	Chronic cholecystitis					15.8	3.73
N8	39	M	Chronic cholecystitis					2.6	2.73
N9	61	F	Chronic cholecystitis					6.4	1.11
N10	61	M	Chronic cholecystitis					<2.0	2.73
N11	39	F	Chronic cholecystitis					9.3	1.01
N12	46	M	Chronic cholecystitis					3	0.76
N13	46	F	Chronic cholecystitis					6.6	0.71
N14	61	M	Chronic cholecystitis					7	0.92
N15	68	M	Chronic cholecystitis					10.2	1.83
N16	55	M	Chronic cholecystitis					6	0.4
N17	39	F	Chronic cholecystitis					<2.0	1.7
N18	68	M	Chronic cholecystitis					21.5	3.31
N19	39	F	Chronic cholecystitis					28.3	2.32
N20	31	F	Chronic cholecystitis					8.4	0.47
